# Direct-Acting Antiviral Agents for HCV-Associated Glomerular Disease and the Current Evidence

**DOI:** 10.3390/pathogens8040176

**Published:** 2019-10-04

**Authors:** Fabrizio Fabrizi, Roberta Cerutti, Giulia Porata, Piergiorgio Messa, Ezequiel Ridruejo

**Affiliations:** 1 Foundation IRCCS Ca’ Granda Ospedale Maggiore Policlinico, 20122 Milano, Italy; roberta.cerutti@policlinico.mi.it (R.C.); Giulia.porata@policlinico.mi.it (G.P.); Piergiorgio.messa@policlinico.mi.it (P.M.); 2 Department of Clinical Sciences and Community Health, University of Milan, 20122 Milan, Italy; 3 Hepatology Section, Department of Medicine, Centro de Educacion Medica e Investigaciones Clinicas Norberto Quirno “CEMIC’’, Ciudad Autonoma de Buenos Aires C1425ASG, Argentina; eridruejo@gmail.com; 4 Hepatology and Liver Transplant Unit, Hospital Universitario Austral, Pilar, Provincia de Buenos Aires B1629AHJ, Argentina; 5 Latin American Liver Research, Educational and Awareness Network (LALREAN), Pilar, Provincia de Buenos Aires B1629AHJ, Argentina

**Keywords:** direct-acting antiviral agents, glomerulonephritis, hepatitis C virus, mixed cryoglobulinemia, proteinuria, sustained virological response

## Abstract

Glomerular disease is an extra-hepatic manifestation of hepatitis C virus infection (HCV) and membranoproliferative glomerulonephritis is the most frequent glomerular disease associated with HCV. It occurs commonly in patients with HCV-related mixed cryoglobulinemia syndrome. Patients with HCV-related glomerular disease have been historically a difficult-to-treat group. The therapeutic armamentarium for HCV-related glomerular disease now includes antiviral regimens, selective or non-specific immunosuppressive drugs, immunomodulators, and symptomatic agents. The treatment of HCV-associated glomerular disease is dependent on the clinical presentation of the patient. The recent introduction of all-oral, interferon (IFN)-free/ribavirin (RBV)-free regimens is dramatically changing the course of HCV in the general population, and some regimens have been approved for HCV even in patients with advanced chronic kidney disease. According to a systematic review of the medical literature, the evidence concerning the efficacy/safety of direct-acting antiviral agents (DAAs) of HCV-induced glomerular disease is limited. The frequency of sustained virological response was 92.5% (62/67). Full or partial clinical remission was demonstrated in many patients (*n *= 46, 68.5%) after DAAs. There were no reports of deterioration of kidney function in patients on DAAs. Many patients (*n *= 29, 43%) underwent immunosuppression while on DAAs. A few cases of new onset or relapsing glomerular disease in patients with HCV successfully treated with DAAs have been observed. In summary, DAA-based combinations are making easier the management of HCV. However, patients with HCV-induced glomerular disease are still a difficult-to-treat group even at the time of DAAs.

## 1. Introduction

More than 71 million people have chronic hepatitis C virus (HCV) infection worldwide. HCV is a major health problem, and long lasting HCV results in chronic hepatitis, cirrhosis, and hepatocellular carcinoma. HCV plays both a hepatotropic and lymphotropic activity and is an important antigenic stimulus for clonal B cell expansion. The detection of circulating cryoglobulins is frequent in patients with chronic HCV (up to 50%) but only 2–3% develop vasculitic symptoms that characterize HCV-mixed cryoglobulinemia syndrome. HCV infection is the main cause of mixed cryoglobulinemia, a systemic vasculitis producing various clinical manifestations which range from the so-called MCS (purpura, asthenia, and arthralgias) to important abnormalities including neurological and renal diseases [1,2]. Rheumatoid factor is consistently elevated in 45–68% of cases, and low complement titers occur in 51–76% of patients. HCV-MCS is more frequent in women than men and affects patients with cirrhosis more commonly than chronic hepatitis. It appears that HCV-MCS is not affected by HCV genotype, viral load, or duration of HCV infection [1,2].

Mixed cryoglobulinemia (MC) represents 60–75% of all cryoglobulinemia and is found in connective tissue disease and infectious or lymphoproliferative disorders (i.e., secondary MC) [3]. After the identification of HCV, it was recognized as the cause of 80–90% of MC. HCV is usually associated with type II MC (which commonly involves an IgM *k* molecule showing rheumatoid factor activity with anti-idiopathic function), although HCV is also associated with type III MC. Around 5–10% of all MCS have no clear etiology. Cryoglobulinemic vasculitis is classified as essential or idiopathic [3].

The incidence of HCV-associated glomerular disease is low and this makes it difficult to conduct clinical trials to control the disease [3]. Renal disease is now considered an important cause of morbidity and mortality in MC vasculitis induced by HCV [3]. Treatment of HCV has made considerable progress with the recent introduction of IFN-free combinations of DAAs which increased dramatically the rates of viral clearance [3]. The purpose of this review is to report the most important data concerning the treatment of HCV-related glomerular disease, an important extra-hepatic complication of HCV infection.

## 2. HCV-Associated Kidney Disease: Histology

Although HCV has been identified as a prominent agent of tubulointerstitial damage in a large series [4], glomerular diseases remain the most frequent kidney disease associated with HCV. The relationship between HCV and glomerular disease has been noted in both the native kidneys and after solid organ transplant (kidneys, liver, or liver/kidney transplant). In the past two decades, a consistent link has been noted between HCV and immune complex (IC) glomerular disease, including membranoproliferative and membranous glomerulonephritis [1,2].

The most common glomerular disease in HCV-positive patients is cryoglobulinemic membranoproliferative glomerulonephritis (MPGN). Other immune complex glomerular diseases associated with HCV are non-cryoglobulinemic GN, membranous nephropathy, IgA nephropathy, immunotactoid glomerulopathy/fibrillary glomerulonephritis (Table 1). Focal segmental glomerulosclerosis and mesangial proliferative glomerulonephritis are additional forms of glomerular disease observed in HCV-infected patients. Polyarteritis nodosa is another uncommon form of kidney disease associated with HCV. It is probably related to the IC deposition in medium-sized muscular arteries leading to ischemic changes in the glomeruli.

We recommend kidney biopsy in HCV-infected patients with clinical evidence of glomerular disease. The course of these HCV-associated nephropathies is characterized by remission and exacerbation phases. However, the long-term outcome remains poorly defined.

## 3. HCV and Kidney-Updated Evidence

HCV plays several activities in numerous organs and systems other than the liver, including kidneys. The kidneys are an important target of the extra-hepatic activity of HCV which causes kidney damage in multiple ways. (1) HCV is a cause of tubulointerstitial nephritis [2], even if this finding needs to be confirmed in further studies (2). HCV leads to glomerular injury particularly in patients with HCV-associated MCS. HCV is an important cause of MCS, a vasculitis involving small to medium vessels (3). Finally, HCV supports the development (or the progression) of chronic kidney disease in the general population, probably by vascular damage at kidney level [5].

A role of chronic HCV infection in the pathogenesis of CKD has been suggested by several studies mostly performed in the last decade. The relationship between HCV and CKD is complex and shows bidirectional nature. Patients on maintenance dialysis remain at risk for HCV infection due to nosocomial transmission of HCV and have a high prevalence of HCV infection. On the other hand, a significant frequency of HCV infection has been noted in patients with renal insufficiency at pre-dialysis stage suggesting a role of HCV in developing CKD [3].

A good number of large databases have addressed the relationship between HCV and chronic kidney disease. According to a recent systematic review and meta-analysis, aggregating results of longitudinal studies (*n *= 15 studies, *n *= 2,299,134 unique patients) demonstrated a correlation between positive anti-HCV serologic status and increased frequency of CKD, the weighted estimate for adjusted HR with HCV across the surveys, 1.54 (95% CI, 1.26; 1.87, *p* < 0.001). Between-study heterogeneity was found (*Q*-value by chi-squared test, 500.3, *p *< 0.0001) and this hampered more definitive conclusions [5]. An accelerated endothelial damage at kidney level (renal arteries and arterioles) has been cited to explain the detrimental role of HCV on the incidence and progression of CKD in the adult general population all over the world. Endothelial dysfunction in HCV-infected patients is in turn supported by oxidative stress, pro-inflammatory cytokines, peripheral or hepatic insulin resistance, or non-alcoholic steatohepatitis (NASH) [5].

HCV and CKD are remarkable public health problems worldwide. The overall mean prevalence of CKD in the general population is around 13.4% (stages 1 to 5) and 10.6% (stages 3 to 5). Traditional risk factors for developing chronic renal disease (diabetes mellitus, arterial hypertension, smoking, weight overload) do not explain completely the current prevalence of chronic kidney disease in the adult general population of industrialized world. Chronic infections by hepatotropic viruses (hepatitis B and hepatitis C viruses) or human immunodeficiency virus (HIV) are non-traditional, but modifiable causes of chronic kidney disease [6,7]. The adverse impact of chronic HCV on chronic kidney disease suggests the antiviral therapy of all HCV-infected patients, regardless of their stage of liver and kidney injury [3].

## 4. Treatment of HCV-Related Glomerular Disease: Historical Perspective

The identification of HCV and a better comprehension of the pathophysiological mechanisms of diseases have provided the possibility to treat HCV-associated glomerular disease with various approaches. (1) Antiviral therapy has been given with the idea that the underlying infection promotes the synthesis of immune complexes and resultant glomerular disease; (2) Nonspecific immunosuppressive agents (corticosteroids, cyclophosphamide, azathioprine, etc.) lowering glomerular inflammation have been adopted by several authors; (3) An additional choice is given by rituximab (RTX), which selectively targets B cells and causes depletion of B cells. Thus, RTX diminishes the synthesis of IgM with rheumatoid factor (RF) activity that bind HCV-IgG immune-complexes; (4) RBV mono-therapy proved to be effective and safe in the treatment of HCV-induced glomerular disease post liver transplant due to its immunomodulatory properties [3]; and (5) Symptomatic drugs such as diuretics, anti-hypertensive, or antiproteinuric (ACEIs or ARBs) drugs should be considered.

Unlike HBV and HIV, HCV infection can be completely and permanently treated by antiviral medications as HCV shows no long-term reservoir in the body. The development of sustained virological response (SVR), which is defined as the removal of HCV RNA from serum for 12 weeks after antiviral therapy ended, is the definitive cure of HCV infection. Johnson et al. were the first authors to report a cohort of patients with HCV-associated glomerular disease who received antiviral therapy (mono-therapy with conventional IFN for two to 12 months). Four patients achieved reduction of serum HCV RNA and contemporary reduction of proteinuria [8].

In the report of Rossi et al., three patients with HCV-associated cryoglobulinemic glomerulonephritis received IFN-based therapy (pegylated IFN and RBV) for 12 months. The patients underwent percutaneous kidney biopsy at baseline and repeated kidney biopsies 14–26 months after antiviral treatment ended. They found lowered glomerular inflammation, and renal vasculitic lesions occurred in one patient only at baseline and disappeared after treatment. Subendothelial deposits disappeared in two of three patients and the number of mesangial cells and monocytes was reduced in all [9].

Subsequent studies were performed mostly in patients with HCV-associated cryoglobulinemic vasculitis (many with kidney involvement) [1,10]. All the authors observed that the elimination of HCV RNA from serum after antiviral therapy gave improvement of clinical abnormalities (including renal disease). These reports gave emphasis to the fact that IFN-based therapies are less effective and more toxic in individuals with HCV-associated MCS in comparison with the general population infected with HCV.

A systematic review and meta-analysis concerning mono-therapy with conventional or pegylated IFN for HCV-MCS in the non-transplant setting was undertaken (*n *= 11 clinical studies, *n *= 235 unique patients). It showed that the weighted estimate of frequency of sustained virological response was 15% (95% CI, 8–22%). The frequency of patients who interrupted antiviral therapy was 3.4% (most patients experienced minor adverse events which did not require discontinuation of therapy). An excellent relationship between virological and clinical response was found (weighted K = 0.72). Cirrhosis (*p *< 0.04), kidney involvement (*p *< 0.07), and arthralgias (*p *< 0.04) at baseline showed negative impact on viral response [11].

The efficacy and safety of combined antiviral therapy (pegylated IFN plus RBV) for the treatment of HCV-MCS in non-immunosuppressed population were also evaluated. 10 clinical studies (*n *= 300 unique patients) were retrieved. The frequency of kidney involvement at baseline ranged between 4% and 39%. The overall estimate of the frequency of SVR was 42% (95% CI, 31–54%). The pooled estimate of frequency of patients interrupting (or dose reducing) antiviral therapy was 15% (95% CI, 8–21%). A good correlation between viral and clinical response (weighted K = 0.63) by a meta-analysis at individual level on a subgroup of studies (*n *= 3, *n *= 74 unique patients) was noted [12].

## 5. Antiviral Treatment of HCV with DAAs and Renal Impairment

**Figure 1:**
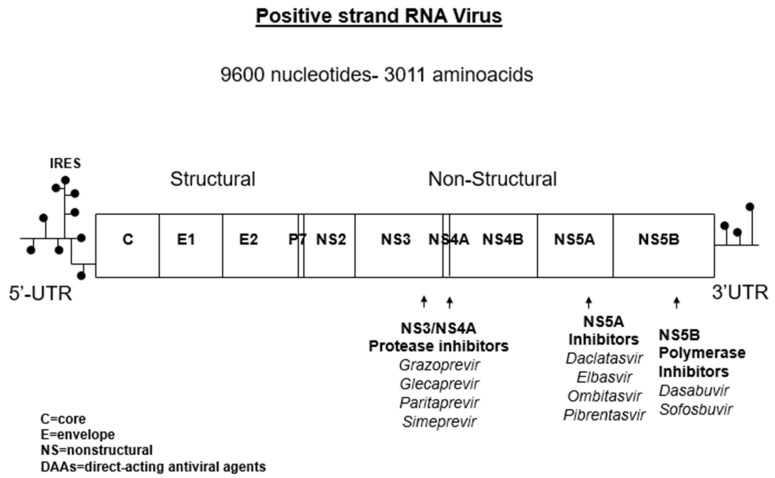
HCV RNA genome structure and targets for DAAs.

Direct-acting antiviral agents are molecules that affect the non-structural proteins of HCV and block HCV replication. Three groups of DAAs are now available for the treatment of HCV. These include NS3/4A protease inhibitors, NS5A inhibitors, and NS5B polymerase inhibitors [Figure 1]. The first-generation NS3/4A protease inhibitors (telaprevir and boceprevir) have been introduced for the treatment of HCV in combination with pegylated IFN and RBV (2011). The triple combined antiviral regimens (IFN/RBV/telaprevir or boceprevir) increased treatment success, but were poorly tolerated [13].

The standard of care is now given by IFN-free combinations of DAAs. These regimens are very effective with SVR rates around 95%, irrespective of the viral characteristics of HCV. Tolerance to DAAs is very good- the frequencies of serious adverse events (AEs) are less than 10% and early discontinuation rates less than 5% [3].

The American Association for the Study of Liver Diseases (AASLD) and the Infectious Diseases Society of America (IDSA) have issued clinical guidelines in order to recommend appropriate combinations of DAAs in patients with kidney impairment. These guidelines have been updated recently (September 2017) [14]. All patients with eGFR ≥ 30 mL/min/1.73 m^2^ can be treated with any licensed DAA-based regimen. As listed in Table 2, three DAA-based regimens have been recommended for patients on maintenance dialysis or those with eGFR < 30 mL/min/1.73 m^2^. Sofosbuvir is the key drug of many DAA-based combinations that are currently available, but is not suggested in patients with eGFR < 30 mL/min/1.73 m^2^ due to the accumulation of its active metabolite GS-331007. On the grounds of large surveys, we observed remarkable deterioration of kidney function following therapy with sofosbuvir-based regimens [15]. The daily fixed-dose elbasvir/grazoprevir regimen is recommended for the treatment of genotype 1 infection in patients with severe renal dysfunction. The C-SURFER study showed that the elbasvir/grazoprevir combination is effective in patients with HCV genotype 1 infection and CKD stage 4/5 [16]. However, elbasvir/grazoprevir shows efficacy in patients with intact kidneys and HCV genotype 1 and 4. Thus, it has been extrapolated that elbasvir/grazoprevir is efficacious in patients with CKD stage 4/5 and HCV genotype 4. The EXPEDITION-4 trial found that the pangenotypic NS3/NS4A protease inhibitor glecaprevir and the pangenotypic NS5A inhibitor pibrentasvir provide a very high rate of viral response in patients with HCV genotype 1, 2, 3, 4, 5, and 6 infection [17].

**Figure 2:**
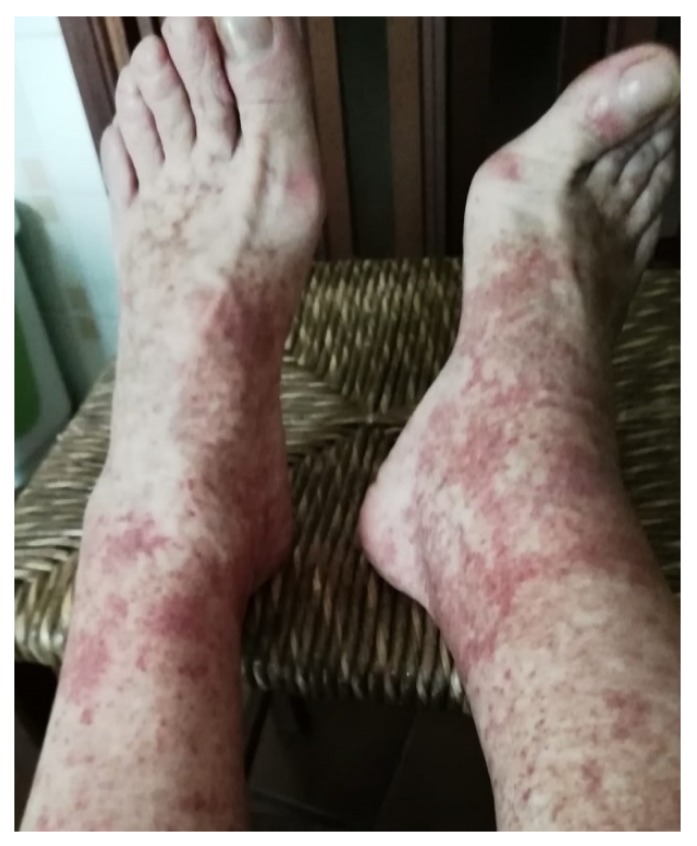
Purpuric manifestations in the legs after successful therapy with DAAs.

Finally, we suggest that serum creatinine and urinary abnormalities should be monitored with accuracy in all patients after antiviral treatment with DAAs. New–onset or relapsing glomerular disease has been noted by some authors in patients who achieved SVR with regimens based on DAAs [18,19,20]. Figure 2 shows purpuric manifestations in the lower limbs in a patient with HCV-related cryogobulinemic patient who received DAAs. After DAAs, the patient obtained SVR but cryoglobulins remain detectable in serum. The patient suffered from frequent and spontaneous episodes of purpura over the observation period (i.e., three or four times a year). Purpura disappeared spontaneously in most cases.

## 6. Antiviral Treatment of HCV (DAAs) for HCV-Related Glomerular Disease

As shown in Table 3, reports regarding DAA-based regimens for HCV-related glomerular disease are limited [21,22,23,24,25,26,27,28,29]. Collectively, the frequency of SVR was 92% (62/67). The rate of complete clinical response after DAAs was 32.8% (22/67). A number of patients required immunosuppression while on therapy with DAAs (*n *= 29, 43%). Some patients received immunosuppression after antiviral therapy ended [28].

The evidence shown in Table 3 is incomplete. As an example, the details on kidney disease (including kidney biopsy) at baseline and after antiviral therapy ended were frequently lacking, the information on type and dose of immunosuppressive therapy before, during and after antiviral therapies was not extensively given. The low incidence of HCV-related glomerular disease clearly hampers the conduction of RCTs or observational studies with large size.

In addition to the studies reported above, small patient series (reviewed in [29]) and some case reports have been published. At our knowledge, at least 11 patients with HCV-associated glomerular disease experienced clinical benefit after completing therapy with DAAs [29,30,31,32,33,34].

It is well known that the removal of HCV RNA from serum is frequently accompanied by clinical improvement of glomerular abnormalities. However, the clinical remission was incomplete or lacking in many patients (Table 3). HCV-related glomerular disease is not easily controlled by antiviral therapies, particularly in the case of proliferative or sclerotic injury. Also, information on DAAs efficacy in HCV-associated cryoglobulinemic vasculitis is disappointing due to the inability of these drugs to inhibit the immune-mediated process once it has been triggered. In other words, even with viral eradication, the course of mixed cryoglobulinemic disease or cryoglobulinemic glomerulonephritis is difficult to predict.

The natural history of HCV-induced mixed cryoglobulinemia is clinically variable. Some patients have an indolent course while others develop vasculitic lesions in various organs including kidneys. The exacerbation of extra-renal symptoms is often associated with a flare-up of kidney disease, but can occur independently. No link between serum complement or rheumatoid factor levels and the course of cryoglobulinemic glomerulonephritis has been noted. However, the clearance of HCV RNA from serum gives clinicians the opportunity to adopt immunosuppressive therapies with no concern on HCV replication.

As shown in Table 3, while DAA use is common, a small number of patients have been treated with IFN and/or RBV and DAAs. Based on the known side effects of IFN and particularly the hematologic side effects of RBV [3], we suggest the adoption of IFN-free/RBV-free antiviral therapies for HCV-induced glomerular disease.

Antiviral therapies will evolve in the near future with the introduction of DAAs provided with reduced treatment duration and lower prices; this will be an additional advance in the management of HCV-related glomerular disease as these patients frequently receive multiple medications due to several comorbidities related to HCV.

## 7. Immunosuppressive Agents for Treatment of HCV-Related Glomerular Disease

As mentioned above, the therapeutic armamentarium for HCV-related glomerular disease includes numerous agents in addition to antivirals. Immunosuppression has often been employed in the past as first-line therapy in cryoglobulinemic vasculitis (particularly when kidney involvement is severe) despite the possibility of an exacerbation of infections.

Table 4 shows that the management of HCV-related glomerular disease is based on the clinical manifestations of glomerular disease. Patients showing a cryoglobulinemic flare or severe glomerular damage induced by HCV should be treated first with immunosuppressive agents (with rituximab as first-line agent) with or without plasma exchange. Current practice is to treat with immunosuppressants until the acute phase of disease is overcome. After that, treatment with DAAs is suggested. The initial approach includes immunosuppressive therapy alone or combined therapy (immunosuppressive drugs and DAAs), according to the decision of the clinician.

Patients with mild or moderate forms of HCV-induced glomerular disease (stable kidney function and/or non-nephrotic proteinuria) should be treated with DAAs. Patients with HCV-induced glomerular disease who are resistant or intolerant to DAA therapies should be given immunosuppressive agents. In all cases, treatment with antiproteinuric agents such as angiotensin-converting enzyme inhibitors and/or angiotensin-receptor blockers should be considered to achieve a maximal antiproteinuric response. Diuretics and anti-hypertensive agents should be given where clinically requested.

## 8. Rituximab for Treatment of HCV-Related Glomerular Disease

RTX is currently the first-line immunosuppressive agent for HCV-related glomerulonephritis [3]. RTX is a monoclonal antibody directed at the lymphocyte membrane-protein CD20 and it gives selective depletion of CD20 positive B lymphocytes. Thus, RTX abolishes IgM production and cryoglobulins. Two RCTs have demonstrated the superiority of rituximab mono-therapy as compared with conventional immunosuppressive therapy (i.e., corticosteroids, azathioprine, cyclophosphamide, and plasma exchange) for the management of HCV-associated cryoglobulinemic vasculitis in individuals for whom prior IFN therapy failed to give disease remission, or in patients who were not eligible for IFN therapy [35,36]. Of note, only a minority of these patients suffered from kidney involvement. Over 400 patients with mixed cryoglobulinemia have been treated with RTX according to a recent and systematic review of the medical literature [37].

A long-term (mean follow-up, 72.5 months) prospective, single-centre and open label study has been recently published- 31 patients (27 infected with HCV) with symptomatic MC (MC type 2 in 29 patients and MC type 3 in two patients) received RTX [38]. There were 16 patients with diffuse MPGN, 26 patients with peripheral neuropathy, and seven with severe skin ulcers. RTX was given at a dose of 375 mg/m^2^ according to a ‘4 plus 2 protocol’ (four doses at 1, 8, 15 and 22 days, followed by two further doses 1 and 2 months later). Five individuals also received intravenous therapy with corticosteroids (500 mg/day of methylprednisolone for three consecutive days). No additional immunosuppressive medications or antiviral agents were administered. We observed complete disappearance of pre-treatment manifestations in all patients with purpuric changes and non-healing vasculitic ulcers, and in 80% of peripheral neuropathies. Cryoglobulinemic nephropathy consistently improved during the observation period, starting from the second month after RTX (serum creatinine from 2.1 ± 1.7 to 1.5 ± 1.6 mg/dL, *p *< 0.05; and 24-h proteinuria from 2.3 ± 2.1 to 0.9 ± 1.9 g; *p *< 0.05). Improvement of cryoglobulinemic serological hallmarks (including cryocrit and low complement C_4_) were observed and no adverse clinical events were noted [38].

We suggest caution about RTX administration as relevant side events have been observed following its use for cryoglobulinemic vasculitis. Prospective data from the AIR (Autoimmunity and Rituximab) registry, which includes data on patients who received RTX off-label, have reported that among patients (*n *= 23) having non-viral cryoglobulinemia vasculitis on RTX, side effects occurred in almost half of the patients (*n *= 11), including severe infections. Episodes of infection had been observed mainly in a subgroup of patients (age > 70 years, essential type II MC, GFR < 60 mL/min/1.73 m^2^, and simultaneous high dose corticosteroids) and were fatal in some cases (*n *= 3) [39]. Significant kidney impairment at baseline, advanced age and contemporary intravenous high-dose corticosteroids were recorded in our patients who had sepsis following RTX administration [40]. One single dose of RTX for gastric lymphoma resulted in the occurrence of cholestatic hepatitis in a renal transplant (RT) recipient with chronic HCV. An enormous increase of HCV RNA levels after RTX administration was noted and the patient died due to bacterial pneumonia [41].

## 9. Non-Selective Immunosuppression for HCV-Related Glomerular Disease

Plasma exchange has been considered for many years the option of choice for MCS, with or without kidney involvement, with the aim of obtaining clearance of circulating cryoglobulins from the plasma and lowering the deposition of ICs to the kidneys. Intravenous or oral administration of corticosteroids has been given to alleviate inflammation at glomerular level. However, steroids support progressive liver disease and replication of HCV [3]. Cyclophosphamide has been selected to improve glomerular disease by lowering stimulation of B lymphocytes and cryoglobulin synthesis. However, liver toxicity is a serious adverse event that limits its use [42]. Mycophenolate mofetil inhibits proliferation of T- and B-lymphocytes, thereby suppressing antibody synthesis and cell-mediated immune responses. Evidence on mycophenolate mofetil for HCV-associated glomerular disease is extremely limited [43].

## 10. Conclusions

The present review shows that antiviral therapy with DAA combinations provides high rates of virological response even in patients with HCV-related glomerular disease. Tolerance was satisfactory with no serious adverse events. Viral response is accompanied with some clinical benefit and immunosuppressive therapies are required in many cases. Patients with HCV-induced glomerular disease are still a difficult-to-treat group even at the time of DAAs. Whether the advent of newer DAAs will reduce the need of immunosuppressive drugs for HCV-related glomerular disease is a topic of active research.

## Figures and Tables

**Table 1:** Kidney disease associated with HCV: pathogenesis and clinical presentation.

Kidney Disease	Pathogenesis	Clinical Presentation
Cryoglobulinemic membranoproliferative GNs	Subendothelial and mesangial cryoglobulin deposits; mesangial deposits of immune complexes (HCV viral antigens, Ig and complement)	Nephritic or nephrotic syndrome
Noncryoglobulinemic membranoproliferative GN	Mesangial deposits of immune complexes (HCV viral antigens, Ig and complement)	Nephritic or nephrotic syndrome
Mesangial proliferative GN	Direct activity of HCV on mesangium	Proteinuria and/or haematuria
Membranous nephropathy	Subepithelial deposits of immune complexes	Nephrotic syndrome
Berger’s disease (IgA nephropathy)	Mesangial deposits of immune complexes	Nephritic syndrome, isolated proteinuria and/or haematuria
Tubulo-interstitial nephritis	HCV deposition in tubular epithelial (perinuclear areas) and infiltrating cells	Proteinuria
Focal and segmental glomerulosclerosis	Direct injury by HCV on podocytes of epithelial cells	Nephrotic syndrome, isolated proteinuria
Polyarteritis nodosa	Immune complexes in medium-sized muscular arteries	Haematuria and/or proteinuria
Immunotactoid glomerulopathy	Deposits (glomerular capillary wall and mesangium) containing microtubular structures	Nephrotic syndrome, isolated proteinuria and/or haematuria
Fibrillary GN	Mesangial deposits (containing randomly oriented fibrillar material) (fibrils composed of antigen-antibody immune complexes)	Nephrotic syndrome, isolated proteinuria and/or haematuria

**Table 2:** Regimens based on DAAs currently available for treatment of HCV in CKD.

Daclatasvir (60 mg)	CKD stage 1,2,3
Elbasvir/Grazoprevir (50 mg/100 mg)	
Glecaprevir/Pibrentasvir (300 mg/120 mg)	
Ledipasvir/Sofosbuvir (90 mg/400 mg)	
Sofosbuvir/Velpatasvir (400 mg/100 mg)	
Simeprevir (150 mg)	
Sofosbuvir (400 mg)	
Sofosbuvir/Velpatasvir/Voxilaprevir (400 mg/100 mg/100 mg)	
Ritonavir-boosted Paritaprevir/Ombitasvir/Dasabuvir±Ribavirin (PrOD or 3D regimen) (50 mg/75 mg/12.5 mg/250 mg/200 mg)	CKD stage 4,5
Elbasvir/Grazoprevir (50 mg/100 mg)	
Glecaprevir/Pibrentasvir (300 mg/120 mg)	

**Table 3:** Viral and clinical response after antiviral therapy with DAAs in patients with HCV-associated GN.

	DAAs	SVR12	Complete Clinical Response	Partial Clinical Response	Concomitant IS
Gragnani L, et al. (2016) (*n *= 4)	SOF-based regimen	4(100%)	3(75%)	1(25%)	1(25%)
Sise M, et al. (2016) (*n *= 7)	SOF+SIM (*n *= 6) SOF+RBV (*n *= 1)	6(86%)	3(43%)	4(57%)	2(29%)
Saadoun D, et al. (2016) (*n *= 5).	SOF+RBV	4(80%)	0	4(80%)	2(40%)
Sollima S, et al. (2016) (*n *= 5)	SOF+RBV SOF+DCV SOF+SIM 3D	5(100%)	0	1(20%)	0
Emery J, et al. (2017) (*n *= 10)	SOF+RBV SOF+SIM 3D±RBV SOF+LDV±RBV	7(70%)	2(20%)	2(20%)	4(40%)
Saadoun D, et al. (2017) (*n *= 5)	SOF+DCV	5(100%)	3(60%)	1(20%)	NA
Bonacci M, et al. (2018) (*n *= 9)	SOF-based regimen±RBV 3D±RBV SIM+DCV GZR+EBR pegIFN+DAAs FDV+DLR	9(100%)	6(67%)	3(33%)	5(55%)
Fabrizi F, et al. (2018) (*n *= 13)	SOF+RBV (*n *= 6) 3D+RBV (*n *= 4) SOF+LDV (*n *= 1) SOF+DCV+RBV (*n *= 2)	13(100%)	3(23%)	7(54%)	9(69.2%)
Obrisca B, et al. (2019) (*n *= 9)	3D	9(100%)	2(23%)	1(10%)	6(67%)

3D+ribavirin (RBV), ritonavir-boosted paritaprevir/ombitasvir/dasabuvir with or without RBV; EBR, elbasvir; FDV, faldaprevir; GRZ, grazoprevir; LDV, ledipasvir; PegIFN, pegylated interferon; SIM, simeprevir, SOF, sofosbuvir.

**Table 4:** Treatment of HCV-related glomerular disease according to clinical presentation.

Presentation	Treatment
Non-nephrotic proteinuria	DAA-based regimen (Table 1) ACEIs and/or ARBs Diuretics, anti-hypertensive agents
Stable and mild kidney dysfunction (GFR > 30 mL/min/1.72 m^2^)	
Nephrotic syndrome	Rituximab, Plasma-exchange, IV steroids, mycophenolate mofetil DAA based regimen (Table 1) ACEIs and/or ARBs Diuretics, anti-hypertensive agents
Cryoglobulinemic flare	
Rapidly progressive glomerulonephritis	

ACEIs = angiotensin-converting enzyme inhibitors; ARBs, angiotensin-receptor blockers; DAAs = direct-acting antiviral agents; GFR = glomerular filtration rate; iv = intravenous.

## Abbreviations

**ACEIs**	Angiotensin-converting enzyme inhibitors
**AEs**	Adverse events
**ARBs**	Angiotensin-receptor blockers
**CI**	Confidence Intervals
**CKD**	Chronic kidney disease
**DAAs**	Direct-acting antiviral agents
**DCV**	Daclatasvir
**3D**	Ritonavir-boosted paritaprevir/ombitasvir/dasabuvir
**EBR**	Elbasvir
**eGFR**	Estimated glomerular filtration rate
**ESRD**	End-stage renal disease
**FDV**	Faldaprevir
**GRZ**	Grazoprevir
**GN**	Glomerulonephritis
**HBV**	Hepatitis B virus
**HCV**	Hepatitis C virus
**HIV**	Human immunodeficiency virus
**HD**	Haemodialysis
**MCS**	Mixed cryoglobulinemia syndrome
**MPGN**	Membranoproliferative glomerulonephritis
**IFN**	Interferon
**LDV**	Ledipasvir
**pegIFN**	Pegylated interferon
**RBV**	Ribavirin
**RF**	Rheumatoid factor
**RT**	Renal transplant
**RTX**	Rituximab
**SIM**	Simeprevir
**SOF**	Sofosbuvir
**SVR**	Sustained virological response
